# Irish Media Coverage of COVID-19 Evidence-Based Research Reports From One National Agency

**DOI:** 10.34172/ijhpm.2021.169

**Published:** 2021-12-13

**Authors:** Melissa K. Sharp, Zoë Forde, Cordelia McGeown, Eamon O’Murchu, Susan M. Smith, Michelle O’Neill, Máirín Ryan, Barbara Clyne

**Affiliations:** ^1^Health Research Board Centre for Primary Care Research, Department of General Practice, Royal College of Surgeons in Ireland, Dublin 2, Ireland.; ^2^Health Information and Quality Authority, George’s Court, George’s Lane, Dublin 7, Ireland.; ^3^Department of Pharmacology & Therapeutics, Trinity College Dublin, Trinity Health Sciences, Dublin 8, Ireland.

**Keywords:** SARS-Cov-2, COVID-19, Media Coverage, Science Communication, Press Releases, Ireland

## Abstract

**Background:** How research findings are presented through domestic news can influence behaviour and risk perceptions, particularly during emergencies such as the coronavirus disease 2019 (COVID-19) pandemic. Monitoring media communications to track misinformation and find information gaps is an important component of emergency risk communication. Therefore, this study investigated the traditional media coverage of nine selected COVID-19 evidence-based research reports and associated press releases (PRs) published during the initial phases of the pandemic (April to July 2020) by one national agency.

**Methods:** NVivo was used for summative content analysis. ‘Key messages’ from each research report were proposed and 488 broadcast, print, and online media sources were coded at the phrase level. Manifest content was coded and counted to locate patterns in the data (what and how many) while latent content was analysed to further investigate these patterns (why and how). This included the coding of the presence of political and public health actors in coverage.

**Results:** Coverage largely did not misrepresent the results of the reports, however, selective reporting and the variability in the use of quotes from governmental and public health stakeholders changed and contextualised results in different manners than perhaps originally intended in the PR. Reports received varying levels of media attention. Coverage focused on more ‘human-interest’ stories (eg, spread of COVID-19 by children and excess mortality) as opposed to more technical reports (eg, focusing on viral load, antibodies, testing, etc).

**Conclusion:** Our findings provide a case-study of European media coverage of evidence reports produced by a national agency. Results highlighted several strengths and weaknesses of current communication efforts.

## Background

**Key Messages**

** Implications for policy makers**

- In our case study, media coverage appeared to focus more on ‘human-interest’ reports which bring a human face or emotional angle to the presentation of an event, issue, or problem, (eg, spread of coronavirus disease 2019 [COVID-19] by children and excess mortality) as opposed to those that were more technical (eg, focusing on viral load, antibodies, testing, etc). More efforts should be made to increase dissemination of technical reports, especially given the public’s need for factual scientific information in a pandemic.
- Particularly in ‘human-interest’ reporting, there was tension between more enthusiastic responses from the public and politicians versus more cautious interpretations of the evidence from public health and medical experts. Given this difference, efforts should be made to ensure that all perspectives are included within a media report to ensure balanced reporting.
- Selective reporting and variability in the use of quotes from governmental and public health stakeholders changed and contextualized results in different ways than perhaps originally intended in press releases (PRs). This emphasises the need for rigour and accuracy in health research related PRs and the need for better engagement with journalists, who are indirect sources of public education, particularly in a pandemic situation.
- When researchers were quoted, it was largely sourced from quotes provided in the PR. This may have contributed to better reporting in our case study and the use of quote in PRs may be helpful in disseminating accurate information to journalists.

** Implications for the public**

How research findings are presented in the media can influence personal behaviours and perceptions of risk. We analysed nearly 500 coronavirus disease 2019 (COVID-19) related media reports from Irish television, radio, print and online newspapers. All sources discussed the results of nine COVID-19 scientific reports published by Ireland’s Health Information and Quality Authority (HIQA) between April and July 2020. We found that, while media reports generally did not misrepresent scientific findings, they did not include all relevant information and selectively quoted political and public health actors. This selective reporting could have contextualised results in ways not originally intended by researchers. More ‘human-interest’ (less technical) reports also received more attention. The media plays an important role in communicating health research findings. It is important that the public is aware of selective reporting and quoting and that scientists better engage with journalists who are indirect sources of public education, particularly in a pandemic.

The coronavirus disease 2019 (COVID-19) pandemic is a public health emergency causing millions of cases and deaths globally.^1-3^ This pandemic has been accompanied by what the World Health Organization (WHO) describes as an ‘infodemic,’ or an over-abundance of information that makes it hard for people to find trustworthy scientific information when they need it.^4,5^ Reputable information from the WHO and governmental agencies is now competing for attention with an unprecedented proliferation of false information and conspiracy theories,^6-8^ with up to 25%-30% of US and UK samples reporting beliefs in at least one COVID-19 conspiracy theory.^9-11^ Exposure to COVID-19 misinformation can affect personal health behaviours (eg, wearing face masks, social distancing) and may demotivate individuals from seeking out and thoughtfully processing information on COVID-19.^10-15^ Media coverage plays a critical role in shaping public opinion and has the potential to influence behaviour and perceptions of risk.^16-20^ The media plays an important role in the dissemination of findings from health research as public knowledge about COVID-19 is largely acquired through domestic news (TV, print, and digital) and social media platforms.^16,21,22^ In this context, it is important that health research is communicated by researchers, research organisations, and the media clearly and accurately. Furthermore, monitoring media communications to track misinformation and find information gaps is an important component of emergency risk communication.^23^

 Press releases (PRs) are an established communication link between researchers and the media. The content and language in these PRs must be accurate and unbiased as the content within a PR itself represents a large portion of the content of news stories.^24^ High-quality PRs issued by medical journals seem to increase the quality of associated newspaper coverage of health research, whereas low quality PRs might make them worse.^25^ Furthermore, low quality PRs can result in media coverage that amplifies the net effect of reporting errors,^24^ overemphasizes the beneficial effects of treatment,^26^ or generally over exaggerates study findings.^27^

 Analyses of media coverage may provide insights about the ongoing communication strategies for research reports and PRs for this and future pandemics.^28^ Emergency and crisis situations evolve in phases and communication efforts need to adapt and respond according to each phase.^23^ During the initial phases of this pandemic, the need for timely research on a novel disease has caused an influx in scientific outputs in basic science, clinical medicine and public health, in both traditional academic journals and on preprint servers.^29,30^ Unfortunately, early reports have shown that many of these scientific articles are poor quality,^31,32^ at high risk of bias,^33-35^ and with inadequate reporting.^36,37^ This makes accurate communication even more important. Due to this rapid increase in primary research, evidence syntheses have become more common and previously underutilized methodologies, like rapid reviews, have been increasingly popular.^38^ With more focus and weight given to evidence syntheses needed to inform public policies and responses to COVID-19, it is important to explore how these reports are being communicated through PRs and in the media as there is little research directly tracing the transformation of PRs from public health agencies into pandemic news coverage.^39^ Therefore, this study aims to explore the breadth and content of print and broadcast media coverage of PRs and associated research reports issued by one national agency in the initial phases of the COVID-19 pandemic. The specific objectives were to describe:

1. Which topics received the most traditional media coverage?
2. Whether traditional media coverage reflected the key messages of nine selected COVID-19 evidence based research reports and associated PRs?
3. How and which ‘key players’ (political, public health, and national agency representatives) were quoted?

## Methods

###  Data Sources

 The COVID-19 Evidence Synthesis Team within the Health Information and Quality Authority (HIQA) in Ireland produces a range of evidence-based reports on a broad range of public health topics related to COVID-19.^40^ These reports arise directly from questions posed by policy makers and clinicians supporting Ireland’s National Public Health Emergency Team (NPHET). Following international methodology guidance^41,42^ and standardised protocols, reports have thus far included rapid reviews, a database of public health guidance, and epidemiological analyses. Findings from these reports informed the national response to the COVID-19 pandemic in Ireland and influenced international health policy and clinical and public health guidance.^43-47^ These reports covered questions such as those relating to the clinical course and epidemiology of COVID-19, transmission via asymptomatic carriers and in children, screening and diagnostic testing, and public health policies.^40^ To facilitate timely dissemination beyond traditional publication in peer-reviewed journals, these reports are made available on the HIQA website^40^ and social media platforms,^48-51^ and are sometimes accompanied by PRs.

**Figure 1:**
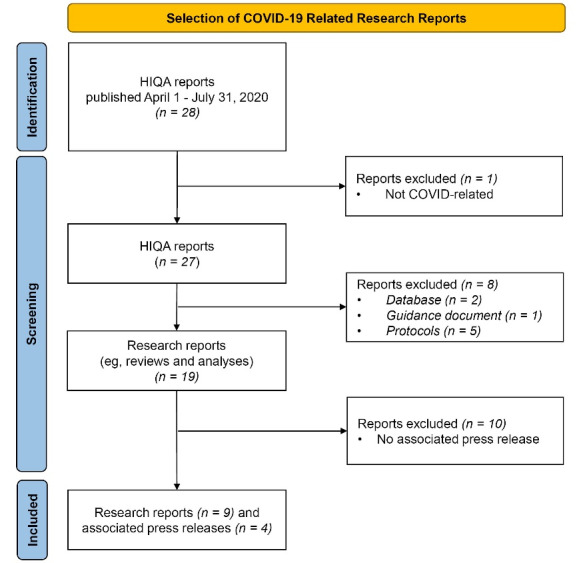
Research Report Flow Diagram. Abbreviations: COVID-19, coronavirus disease 2019; HIQA, Health Information and Quality Authority.

 Data obtained for this cross-sectional study focused on publicly available HIQA reports and PRs published during the initial phases of the pandemic between April 1 and July 31, 2020.^40^ During this time period, the HIQA team published 28 reports and outputs (protocols, public health databases, and guidance documents), 27 of which were related to COVID-19. Nineteen of these were evidence summaries, rapid reviews and health technology assessments (HTAs), and original analyses. Of these 19 items, nine reports were disseminated to the media via accompanying PRs (Figure 1). The titles and references for the eligible reports and PRs are available in Table 1 alongside their ‘key messages,’ the coding of which is described in the analysis section below. We chose to focus on these nine reports as there were active efforts to communicate the information to the press. Each PR was disseminated to Irish national and regional media via email, published on www.hiqa.ie, and promoted across HIQA’s social media channels, in line with how HIQA’s PRs are normally issued. These PRs were then covered by online news sources, national and regional print media, and through broadcast news (eg, radio and television).

**Table 1:** Key Messages From Press Releases and Research Reports

**PR **	**Research Report and Key Messages**
**PR1:** HIQA publishes COVID-19 evidence summaries to support work of the NPHET. *April 1, 2020.* • Announcement of HIQA’s support role to governmental and public health bodies. • Note: individual study findings not reported within PR.	Evidence summary for COVID-19 viral load over course of infection^56^ • The evidence to date suggests a somewhat consistent trajectory of the viral load of SARS-CoV-2 over the course of the disease, peaking around the time of symptom onset. The virus appears to be detectable during the asymptomatic and pre-symptomatic phases, and for around two weeks from symptom onset. • Concerns have been raised about the potential for faecal-oral transmission of SARS-CoV-2, particularly among children. Evidence summary for natural history of COVID-19 in children^57^ • In general, the presentation of symptoms in infants and children is variable, although most have mild symptoms and many are asymptomatic. • The incubation of COVID-19 may be longer in children than in adults and viral shedding in respiratory and stool specimens may occur for longer in children than in adults. Evidence summary for average length of stay in the intensive care unit for COVID-19^58^ • Median length of stay in ICU has been reported to be approximately seven days for patients who survive COVID-19, and eight days for those who do not survive, with shorter lengths of stay reported in the UK. Evidence summary for spread of COVID-19 by children^59^ • There is currently limited information on how children contribute to the transmission or spread of COVID-19.
**PR2:** HIQA publishes a rapid HTA on COVID-19 diagnostic testing. *April 22, 2020.* • Two types of diagnostic tests for SARS-CoV-2: those that detect the virus/particles (RT-PCR and antigen testing); and those that detect the body’s immune response to the infection such as antibody testing. • Validated antibody tests could be used in seroprevalence studies to assess the proportion of the population that has been exposed to SARS-CoV-2. This information will be useful to inform implementation or easing of public health measures. • Adequacy and duration of immunity as well as the possibility of reinfection are largely unknown.	Rapid HTA of alternative diagnostic testing for coronavirus 2 (SARS-CoV-2)^60^ • Diagnostic tests for SARS-CoV-2 can be broadly grouped into two categories: those aimed at detecting the virus and those that detect the body’s immune response to the infection (past exposure to the virus). Real-time PCR is the preferred method to detect SARS-CoV-2 RNA and to confirm acute infection early in the clinical course of COVID-19 disease. Antigen detection tests could be used to supplement current laboratory-based real-time RT-PCR case detection. • The primary role of antibody tests is likely to be as part of well-constructed seroprevalence studies to model the course of the pandemic and inform the public health response. • While the use of antibody tests to provide ‘immunity passports’ has been proposed in the literature,little is known about the adequacy of the immune response or the duration of immunity, and so it is not known if reinfection can occur.
**PR3:** HIQA publishes four evidence summaries to support national response to COVID-19. *May 13, 2020.* • While the evidence is limited, it appears that children are not substantially contributing to the spread of COVID-19 in their household or in schools. • As yet, it is also not certain if antibodies are transferred from mother to the child in the womb via the placenta. • Antibodies against SARS-CoV-2 develop soon after infection. There is no long term evidence of immunity. • It is not yet possible to determine if reinfection is possible following recovery from COVID-19.	Evidence summary for spread of COVID-19 by children^59^ (*Update*) • From the small number of studies identified, it appears that children are not, to date, substantially contributing to the household transmission of SARS-CoV-2. From one study, SARS-CoV-2 transmission in children in schools is also very low, however the evidence remains limited. Evidence summary for placental transfer of antibodies^61^ • Passive transfer of antibodies from mother to infant cannot be confirmed. Evidence summary of the immune response following infection with SARS-CoV-2 or other human coronaviruses^62^ • The median time to antibody detection following symptom onset ranged from five to 13 days for IgM and 12 to 14 days for IgG. SARS-CoV-2-specific IgG antibodies were detected in all individuals after approximately two weeks; however, the adequacy or duration of this response is not yet known. • The full duration of the immune response is unknown. • Ten studies that investigated the association between severity of initial disease and immune responses found inconsistent findings. Evidence summary for the infectiousness of individuals reinfected with COVID-19^63^ • The evidence for whether individuals reinfected with SARS-CoV-2 or other human coronaviruses are infectious is currently inconclusive.
**PR4:** COVID-19 causes 13% increase in deaths in Ireland between March and June 2020 – HIQA. *July 3, 2020.* • Based on an analysis of the death notices reported on RIP.ie since 2010, there is clear evidence of excess deaths occurring since the first reported death due to COVID-19 in Ireland. • However, the number of excess deaths is substantially less than the reported 1709 COVID-19-related deaths over the same period. • In the last four weeks of the analysis, we have seen a reversal of that trend with fewer deaths than expected	Analysis of excess all-cause mortality in Ireland during the COVID-19 epidemic^64^ • Based on the deaths notices reported at RIP.ie, there is clear evidence of excess mortality occurring since the first reported death due to COVID-19 in Ireland. • The officially reported number of COVID-19 deaths for the same period was 1709. Therefore, the estimated excess mortality is less than the officially reported COVID-19-related mortality by 637 cases. The officially reported COVID-19 deaths may overestimate the true burden of excess mortality specifically caused by COVID-19. • The excess mortality observed at the peak is now being followed by a period of decreased mortality as date of death for individuals who would ordinarily have died during this time may have occurred earlier than expected.

Abbreviations: PR, press release; COVID-19, coronavirus disease 2019; HIQA,Health Information and Quality Authority; SARS-CoV, severe acute respiratory syndrome coronavirus; NPHET, National Public Health Emergency Team; HTA, health technology assessment; RT-PCR, Reverse transcription polymerase chain reaction; IgM, immunoglobulin M; IgG, immunoglobulin G; ICU, intensive care unit.

 As a way for HIQA to monitor its media coverage, Rue Point Media was contracted to track mentions of HIQA in Irish news sources.^52^ Rue Point uses a combination of OPoint,^53^ TVEyes,^54^ and an internal search software based on the Java library Lucene.^55^ They search headlines, byline, body text, and captions for search terms based on the brief sent to them by HIQA which outlines relevant words (see Supplementary file 1). Tracking resulted in a data corpus of 523 PDFs of ‘traditional’ media sources potentially related to a report published within our time period. Each source contained a header with standard summary data (provided by Rue Point) such as publication title, publication date, link to the original source (if applicable), and reach (audience). Each source included written and audio-visual text (ie, transcribed from radio sources or soundbites) and could include iconic texts (ie, photographs or images) and hyperlinks. Media sources were eligible for inclusion if they covered at least one of the nine eligible reports; there were no other criterion. All sources were in English or Irish; Irish sources were translated into English by a native speaker. When individual sources are quoted in the results, their code is denoted in [brackets]. Social media sources were not included in this analysis.

###  Analysis

 Prior to viewing media sources, ‘key messages’ (ie, main points) from each publicly available PR and research report were proposed by one author (MKS) to develop the initial coding schema. After several iterative discussions with another researcher (BC), a final scheme was applied (Table 1). We performed a summative content analysis^65,66^ aimed at (1) identifying and quantifying which reports, messages, and stakeholders were present in the coverage to locate patterns in the data (ie, the what and how many;) and (2) further exploring and examining the patterns in the data identified through the first stage (ie, the why and how). This approach combines a quantitative analysis of manifest content and qualitative analysis of latent content to study a phenomenon in an unobtrusive and nonreactive way.^65,66^ The content analysis of the media sources aimed to understand how key messages from each PR and report were reported and framed in comparison to others as media coverage can influence the public’s behaviours and perception of risk.^16-20^ All quantification coding focused on the manifest content (ie, information that is readily understandable at face value) and was applied at the phrase level.^67^ After data was ‘segmented’ by which reports and key messages they covered, data was grouped and categorized to identifying particularly prominent or salient reporting/themes.

 PDFs of the news items were imported into NVivo version 12^68^ for content analysis and Microsoft Excel was used for organizing individual line data containing characteristics of each source (title, publication outlet, publication date, etc) and for descriptive statistics and quantitative coding. One reviewer (MKS) screened each item for eligibility to determine whether it discussed at least one of the nine reports covered in the four PRs (Table 1). After screening, a conceptual (ie, focused on the existence and frequency of concepts) quantitative content analysis was performed (by MKS, an ‘impartial third party’) on the text from each eligible news source. To promote good intracoder reliability (ie, consistency in how the same person codes data at multiple time points) all sources were double-coded and the coding was continually discussed with a content expert (BC). This was deemed sufficient as content analysis promotes the trustworthiness of findings by selecting the most suitable meaning unit (coding at the phrase level), including quotations throughout reporting of results, and through dialogue between researchers.^69^ We also quantified which key players – HIQA researchers, public health specialists (ie, the (Deputy) Chief Medical Officers, CMOs), and politicians (ie, the Taoiseach- prime minister) – were quoted talking about results. Of note, during the reporting period, the CMO took temporary leave and the deputy CMO became interim CMO. Furthermore, on June 27, 2020, a new Taoiseach was appointed and the former Taoiseach Mr. Leo Varadkar was appointed as Tánaiste (Deputy Prime Minister). Sources published after this date (related to the fourth PR) continued to quote him and not the new Taoiseach.

## Results

###  Descriptive Characteristics of Full Dataset 

 After screening for eligibility, 35 sources were excluded. The remaining 488 sources were coded for key messages covered in the research reports and PRs (Table 1). Of the 488 eligible sources, 35 were ‘redundant’ (ie, an identical news story is reported across sources); thus, we had 453 unique items with an average reach (audience) of 174 817. Broadcast media was the largest data source (Table 2). Respectively, of the 488 sources included, PR1 was discussed in 16 (3%) sources, PR2 in 33 (7%) sources, PR3 in 214 (44%) sources, and PR4 in 225 (46%) sources. The Prime Minister (*Taoiseach*) was quoted in 109 sources (22%), the Deputy and CMOs were quoted in 158 sources (32%), and HIQA researchers were quoted in 180 sources (37%). A majority (58%) of quotes from HIQA researchers came from those given in the PRs themselves.

**Table 2:** Media Coverage April 1 to July 31, 2020

**Platform**	**April**	**May**	**June**	**July**	**Total**
National print	1	19	1	12	33
Regional print	3	3	4	6	16
Online	27	88	10	66	191
Magazine	0	3	1	0	4
Broadcast	16	86	1	141	244
**Total**	47	199	17	225	488

**Figure 2:**
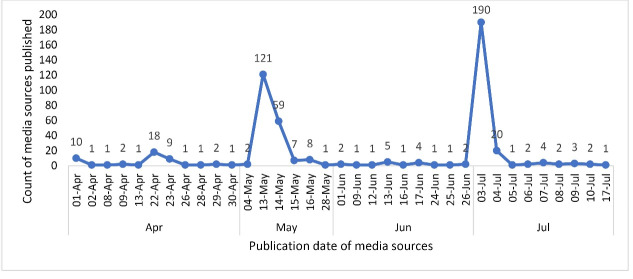
Media Coverage of “Health Information and Quality Authority” Evidence Outputs April 1 – July 31, 2020.

 The third press release (PR3), published on May 13 (covering the reports on spread by children,^59^ placental transfer of antibodies,^61^ immune response,^62^ and reinfection^63^) and PR4 from July 3 (the analysis of excess all-cause mortality^64^) by far received the most coverage (Figure 2). Media coverage was overwhelmingly reported on the day of and day after the PR was published (Figure 2). As PR3 and PR4 received the most media coverage, the results presented focus largely on those.

###  Key Messages and Actors Presented for Each Press Release 

####  Press Release 1

 Briefly, although the first press release (PR1) included links to four completed research reports, it did not actually discuss the results or findings of the reports; it largely introduced HIQA’s work to support NPHET. Accordingly, of the 16 sources covering PR1, over 80% (n = 13) discussed this new role. Of the four reports included in PR1, the one focused on the average length of intensive care unit stay received the most attention (n = 12). The summaries for viral load and spread by children were covered by one source each whereas the report for the natural history of COVID-19 in children received no coverage. Coverage was relatively aligned with the key messaging of the reports with no further comments added from political, public health, or medical actors. Some sources from this time period also discussed the upcoming rapid HTA (discussed in PR2) as Ireland was promoted as being *‘the first European country to carry out such an assessment’ *and *‘European leaders in that regard’ *[005].

####  Press Release 2

 The second press release (PR2) described the publication of a rapid HTA of alternative diagnostic testing and was covered by 31 sources. Reporting largely focused on the key message focusing on the type of test (n = 21), followed by discussions about immunity (n = 10), and seroprevalence studies (n = 2); only one source discussed all three key messages (Table 1). The CMO was quoted in 12 (39%) of the sources, largely with neutral statements introducing why the HIQA Rapid HTA was commissioned – *‘as part of understanding the testing landscape’ *[022] – and how *‘testing is a key element to Ireland’s response to COVID­19’ *[024]. HIQA researchers were quoted in 28 of the sources (5 broadcast) although a majority (n = 25) of quotes appear to come from a supplemental interview with a HIQA researcher rather than using direct quotes from the PR itself.

 “*[HIQA Researcher] said there were ‘knowledge gaps with regard to antibody testing,’ and that ‘independent clinical validation of the analytical performance data provided by manufacturers’ of these tests was of critical importance. Right now, it is unclear whether widespread serological testing will provide a means of deciding how and when to reopen economies – even if very accurate tests come onto the market*”[020].

 Taoiseach Leo Varadkar was quoted in one source, straying away from the key message of the report which focused on testing. “*The tests that have been developed, the anti­body tests are not up to standard and you may have seen the HTA produced by HIQA which goes through that in detail*”[050].

####  Press Release 3

 Over two hundred sources (n = 214) covered PR3. Although the PR discussed the results of four reports, media coverage largely focused on the update to the report for spread of COVID-19 by children (n = 201). For the other three reports covered by PR3, 59 (28%) sources discussed the report on immune response, 41 (19%) discussed reinfection, and 23 (11%) covered the placental transfer of antibodies report. The coverage of the placental transfer of antibodies report was aligned with the key message of the report and only involved quotes from HIQA researchers (n = 22), 20 of which came directly from the PR. Whereas the reporting on the immune response report involved some further contextualising of the results with input from the Deputy CMO (10/59) in addition to prominent quoting of HIQA researchers (39/59), a majority of which came directly from the PR (35/39). HIQA researchers were also highly referenced in media coverage of the report on reinfection (36/41); again, largely using quotes from the PR (32/36).

 “*Continued monitoring is needed to assess the adequacy and duration of the immune response for COVID­19,’ she said *[HIQA researcher]*. ‘Evidence for other types of serious coronavirus infections, such as SARS-CoV-1 [ Severe Acute Respiratory Syndrome coronavirus 1], shows that the antibody response is maintained for one to two years after initial infection and decreases thereafter*”[054].

 “*But Dr Ronan Glynn *[deputy CMO]* says that is not necessarily positive evidence just yet. ‘What it is doing is adding to our understanding of issues around immunity,’ said Dr Glynn. ‘Obviously we need to continue to look at that’” *[251].

 Of the 59 sources covering the immune response report, 58 covered the key messages regarding the duration of immunity, 44 (21%) discussed antibodies, and none reported on the relationship between severity of disease and an immune response. The reporting of this report was largely aligned with the key messages, with no policy implications discussed.

 Of the 201 sources covering PR3 (focused on the spread of COVID-19 by children), the Taoiseach was quoted in 104 sources (52%), the Chief and Deputy CMOs were quoted in 26 sources (13%), and HIQA researchers were quoted in 66 sources (33%), largely using quotes from the PR (54/66). Media coverage also further contextualised the report’s results by adding commentary from a number of stakeholders like other healthcare professionals and the public. This was the only report that included a lot of relational dynamics involving key governmental and public health experts, the public, and healthcare practitioners in the debate. Perhaps contributing to this wide dissemination and involvement of many different stakeholders, the report was also published at a time when key policy decisions were being made:

 “*All schools and childcare facilities have been shut since March. Now, as Ireland prepares for a tentative phased reopening plan, the HIQA has found limited evidence that children are not a major contributor to the spread of COVID­19 in Ireland” *[103].

 Among the stakeholders there was wide variation as to the interpretation of the results. Some healthcare professionals hailed the report as ‘*good news*’ [117, 128] while other healthcare professionals, such as Finland’s ‘top epidemiologist’ and an Irish physician, extrapolated beyond the key messages of the report in their commentary.

 “*Referring to recent epidemiological evidence, ‘the risk of a child infecting an adult is not realistic’ he said, ‘opening schools is risk free.’ He was commenting on this week’s HIQA assessment of a number of studies, which indicated that children were not substantially contributing to the spread of the virus. But those studies were limited, as HIQA was at pains to point out. Very little research has been done as yet in this area” *[113].

 “*There’s no medical evidence that children are at high risk of transmitting this virus. In the weeks and months since then, there’s more and more evidence coming that actually children aren’t superspreaders with Covid, they don’t seem to transmit it between each other or to people at high risk” *[121].

 Some journalists themselves, also extrapolated beyond the key messages, exhibiting a general tone of celebration and unwarranted expressions of certainty:

 “*The risk/benefit ratio is weighted on the side of re­starting: the risk is minimal and the benefit is immense. International science agrees mercifully, children are not targets of this horrible virus and are generally spared. Research has established they are far less likely to get it and when they do it is less severe. They are not significant transmitters. One major study found there is not one case of a child transmitting it to an adult. A HIQA study found children were not contributing to COVID-19 spread, either in school or at home. I could go on. It’s all good news. We should be singing Hallelujah and doing backflips up to the school gates. Yet there seems to be a perverse insistence of dismissing unanimous findings that are the key to unlocking children’s lives and freedom” *[112].

 In terms of political actors quoted in media coverage, there were inconsistencies in how the report was interpreted between politicians themselves, and between politicians and public health specialists. The Prime Minister (Taoiseach) was presented as having an optimistic reading of the evidence.

 “*Listening to the Taoiseach yesterday, you would almost be forgiven for getting the lunch boxes ready for back to school... There was a pep to his step in relation to the schools returning yesterday *[135]*.…he hinted that schools could open ahead of September in light of the new evidence” *[074].

 “*I think we still have a lot to learn about this virus and we are learning all the time. ‘But if you take what HIQA has said today and what Mike Ryan of the World Health Organisation has said today, they are very much of the view *[193]* that the emerging evidence is that among the safest things we can do over the next couple of months is to reopen our schools and childcare facilities’” *[063, 072, 193].

 He continued, adding some sentiment to the argument, saying:

 “*I think it wouldn’t be a good reflection on us as a society if we’re the last people who are able to reopen all schools and reopen our childcare facilities *[141, 067].* But we need to make sure we do it safely and work with the education sector and the childcare sector to make sure that is possible” *[067].

 Other politicians, including the Minister for Health were quoted to have more cautious interpretations of the report’s findings.

 “*Minster for Health Simon Harris said that ‘any report that helps us is welcome’ but he added that we were still dealing with a virus that we are learning more about every day and therefore ‘evidence is inconclusive’ *[193]. He* ‘warned the advice about children visiting grandparents remains unchanged’” *[064]. 

 Political *‘enthusiasm contrasted with the caution of chief medical officer’ *[127]as well.When the CMO was asked about the Taoiseach’s remarks at a daily COVID-19 briefing, he said that in *‘broad terms he would not disagree with Mr. Varadkar’ *[141, 066] but he *‘advised against reading too much into evidence from a small number of studies’ *[127]and added that it was* ‘an entirely different thing to conclude in policy terms that we have enough evidence to say that transmission from children does not occur’ *[279, 141] ‘*and that’s not the kind of information parents need to confidently send their children back to school’ *[141]. In contrast to the Taoiseach’s opinion that reopening the schools would be* ‘the safest thing to do’ *[063, 072, 193], the CMO ‘*said he did not anticipate any imminent change on the National Public Health Emergency Team’s advice that schools should not reopen until September’ *[066].He *‘warned the evidence from HIQA is inconclusive *[067],* ‘stressing not enough studies had been conducted to conclude for certain that children do not transmit the disease’ *[066].Further adding that,* ‘the decision as to when schools should reopen cannot be based solely on estimates of the transmission rate’ *[127].

 An opposition party leader discussed these public disagreements as *‘pretty embarrassing…Those comments were subsequently shut down by the chief medical officer. For the public to hear one message coming from the Taoiseach and, a couple of hours later, to hear the opposite message coming from the CMO is not good’ *[116].

 While the voices of members of the public were largely not presented in media coverage, a letter to a paper also urged caution.

 “*Sir, – I was reassured to hear the Taoiseach state that ‘…the emerging evidence is that among the safest things that we can do over the next couple of months is to reopen our schools and to reopen our childcare facilities to allow children to return to education and to return to normal life’ (News, May 13th). If it is true, it is a wonderful development. The reality may be somewhat different. The HIQA report that informed the Taoiseach’s pronouncement clearly states: ‘The five primary studies were of low to moderate quality for their design, as there was a lack of detail as to how cases were selected, what the criteria for testing contacts was, what testing was undertaken and how consistently testing was conducted across all contacts....Two studies had small sample sizes... and three studies had not undergone peer review at the time of writing.’ It might be wiser to wait for more reliable research. – Yours, etc, JOE McKEOWN, Kilkenny” *[132].

 In light of these conversations, it is unsurprising that some of the media coverage highlighted this confusion and that* ‘the experts can’t agree upon how infectious youngsters are’ *[051]. As one radio broadcast succinctly summed up:

 “*I think it’s really quite a significant story because it this is going to affect anyone with children … but anyway that’s not what the papers leading with and most major outlets took an incredibly positive spin on that very small HIQA report even though it doesn’t state the children don’t get COVID-19, just says that there’s no evidence to prove they are super spreaders but it was portrayed as something completely different altogether but anyway” *[142].

####  Press Release 4

 The fourth press release (PR4) was covered by 224 sources in which the former^2^ Taoiseach was quoted in 4 (2%), the CMO was quoted in 111 (50%) and HIQA researchers were quoted 80 (36%). Roughly half of the quotes from HIQA researchers were from the PR (42/80); four of these combined information from the PR and a supplemental interview. The overestimation key message was reported by 83% of the 224 sources (n = 186), excess deaths was reported by 49% (n = 109), and 14% reported on the recent decrease in deaths (n = 32); only 14% of the sources reported all three key messages (n = 31) (Table 1).

 Several clear patterns were evident in the data where the key message focusing on ‘overestimating deaths’ was the clear takeaway from the reporting. In fact, 85 sources (38%) reported only this key message and did not mention the key messages of ‘excess deaths’ (compared to the prior year) nor the ‘recent decrease in deaths.’ This manner of reporting was especially prominent in the broadcast sources (n = 79). In relation to the overestimation of excess deaths, the acting CMO was often portrayed as having *‘defended the recording of deaths after today’s HIQA’s report on mortality which found excess deaths in Ireland from March to June were substantially less than the officially reported figures’* [35 broadcast sources]. Some other sources reinforced this tone by referring to *‘Ireland’s regime of counting’ *[380, 310] and that the ‘*disease death stats error is an undignified disgrace’ *[270]. The CMO made attempts to explain that these numerical differences did not reflect errors, rather that:

 “*HIQA’s report on mortality today demonstrates that we have comprehensively recorded deaths relating to COVID-19 in Ireland by following the recommended WHO approach. ‘We have consistently recorded and published data on all deaths where a person had COVID­19 or was suspected to have COVID­19. This reporting gives us a robust understanding of the impact of the disease in Ireland and continues to inform our response’” *[385].

 Other sources were more balanced in their reporting although still highlighting the overestimation message and heavily caveating the message focused on excess deaths. Nearly all (104) of the sources reporting on excess deaths (n = 109) also reported the message of overestimation.

 “*It found there were 1200 more this year which conflicts with the Department of Health death toll of more than 1700 linked with COVID­19” *[426].

 “*A new report from the Health Information and Quality Authority found that the excess deaths were substantially less than the coronavirus figures. While it caused a 13% increase in deaths here between March and June, the true number may be less than reported. While there were 1200 more deaths during the period, it is less than the 1709 reported COVID-19 related deaths during those months” *[303].

 Explanations for the reasoning behind overestimation were not common, only being discussed in a few sources.

 “[HIQA Researcher]* says it is better to have overestimated rather than underestimated the figure reported nationally… the official figures for 2019 debts this is probably very close to the true figure whereas excess mortality possibly understates mortality from COVID-19” *[347].

 However, the issue did get national attention, being discussed in proceedings in the Dáil (Irish Parliament).

 “*The reason we counted the way we did was that we thought it the right thing to do in terms of saving lives to overcount, because we could then contact­trace suspected cases, not just confirmed cases, which other countries did not do. The excess death figures produced by HIQA tell us a lot, including that we may be one of the few countries significantly revising down the number of deaths during the pandemic period. For some people, they saw the crisis as some sort of competition as to where they were in a league table” *[469].

## Discussion

 This cross-sectional study examined the media coverage of research reports produced by a national agency (HIQA), support to the NPHET’s decision-making and policy recommendations during the COVID-19 pandemic. Within the context of the COVID-19 ‘infodemic,’ PRs of health research from national agencies are likely to be selected for news coverage, given the public appetite for information about the pandemic.^70,71^ Our findings suggest variability in terms of which reports and key messages received the most media attention and to what degree political and public health actors were present in media coverage. This study demonstrated that ‘human-interest’ reports (eg, the spread of COVID-19 by children EO and excess mortality analysis) received more media coverage – aligning with previous research on global media framing of COVID-19 coverage.^20^

 While we did not quantitatively assess the accuracy of content, media coverage was largely aligned with the key messages of the reports. It was reassuring that a majority of the quotes from HIQA researchers came from the PR itself, perhaps contributing to accurate reporting. However, selective reporting and quoting of politicians and the public changed the narratives for the reports focused on the spread of COVID-19 by children and on excess mortality. Contrary to previous work,^72^ media coverage largely did not make inappropriate causal interpretations of the report’s findings. However, this may have been related to the nature of the reports which were primarily reviews as opposed to primary research. On the other hand, we found redundant information across all sources wherein sections of reporting were verbatim across several sources (ie, the PR wording or quotes from a singular interview or press conference). Reporting was reordered and selectively presented. This finding aligns with previous work which found that many news stories covering a specific meta-analysis were verbatim or moderately edited copies from the PR or two related sources.^24^ From an organisational communication perspective, redundant reporting is not a concern so long as the PR is of high quality. Redundant reporting actually results in consistent reporting of the study findings across media sources.

 Reporting issues were more concerned with selective coverage of reports and the quotes used from stakeholders which were interplayed off of each other to create narratives. This finding reflects previous work done during the 2009 H1N1 pandemic which demonstrated journalists’ selectivity in disseminating governmental PRs.^73^ Journalists act as intermediaries by reshaping and reframing messages^73^ creating “‘interpretative packages’ and adding to the ‘issue culture’ of the topic.”^20,74^ Particularly for the media coverage of the report on spread of COVID-19 by children, there was a tension between caution vs. enthusiasm and evidence vs. opinion.

 The reports that received the most media coverage were arguably those which were more ‘human-interest’ focused – those related to children spreading disease and therefore their ability to go to school and the report on excess mortality or death. More technical reports such as those focused on viral load, antibodies, and testing, received much less media coverage. The human-interest frame is a common and prevalent ‘generic’ news frame which brings a human face or emotional angle to the presentation of an event, issue, or problem.^75^ For the communication of scientific findings, this can be a problematic frame to use as, somewhat paradoxically, a single story receiving disproportionate attention, can cause the audience to actually become less interested in the general information about the issue and more critical and tired.^76^ This is a concern as previous work has shown that reader’s need more technical information about COVID-19 such as ‘updated information on disease and treatment,’ ‘transmission mechanism,’ and ‘epidemiology of symptoms, treatment, and prevention.’^77^ The overuse of this frame can also mislead readers to believe that a problem is more severe than it actually is^78^ which can be extremely problematic for the selective reporting of excess mortality which was unbalanced in covering the ‘overestimation’ key message. Despite this contradiction with reader’s knowledge needs, previous research focused on the 2009 H1N1 pandemic demonstrated that news releases are more likely to be selected for news coverage when they focus on emotional appeals, have positive tones, and are framed as a gain.^39^ Frequent human-interest and emotional (fear/scare/hope) reporting has also been found for media coverage in COVID-19.^20^ This could partially explain why the report focused on the spread of COVID-19 by children received so much attention.

 As previously noted, the Prime Minister was quoted in over half of the sources for PR3 and the report on the spread of COVID-19 by children (52%, 104/201), in contrast to the overall prevalence of quoting across all the sources (22%, 109/488). This difference highlights how discussions about this particular report were more political than others in our dataset. It can be problematic to provide more emotion-focused quotes from politicians (than public health experts), as this reporting can appear to question the authority of science and portray scientific reports as a ‘subsidiary body of knowledge.’^79^ Through human-interest reporting featuring politicians, political expertise in managing the health crisis can boosted.^79^ Although we did not assess reader’s perspectives on this reporting, research has shown that public trust in politicians in Ireland is much lower than trust in scientists, health experts, and government health authorities.^81^

 An analysis of US COVID-19 media coverage found that newspaper coverage is highly politicized while network news coverage is somewhat less so. On the other hand, both newspaper and network news coverage are highly polarized with politicians appearing in newspaper coverage more frequently than scientists.^80^ However, this trend did not align with our findings as, when the Prime Minister was quoted in PR3 media coverage (104/201), he was nearly equally represented in broadcast (50/104) and print/online media (54/104). Our results suggest that when non-expert sources are quoted often in coverage, messages may stray away from evidence-focused and become more emotion-based.

 However, it is not uncommon for quotes to be included in news articles to provide further context and elaboration and it lends itself to the concept of journalists as communication intermediaries. A large majority of UK and Dutch news articles discussing peer-reviewed health-related research included quotes from the original study authors but only 7.5% and 7.0% respectively contained a ‘new quote’ from an expert source not included in the PR.^82^ Our study had far larger representation from the Deputy and Chief CMOs (32%) and HIQA researchers (37%) in the media coverage. The inclusion of the external ‘expert quotes’ may have been a contributing factor into why the reporting of key messages were largely accurate and generally did not exaggerate the causality of findings.^82^

 The four PRs that we included also demonstrate several different public health messaging concerns that should be taken into consideration for public bodies who communicate the results of evidence syntheses. Firstly, all important evidence should be available in the PR, reducing any additional steps or barriers to access the information or interpret scientific outputs. In our corpus, it was extremely rare that media reporting would include information that was in the research report and not in the PR. Previous work has shown that approximately one-third of coverage was derived largely or wholly from the PR, only 14.4% went beyond a secondary source.^24^ Related to this, including brief supplemental public health and epidemiological education within PRs may be beneficial as our coverage of PR4 demonstrated some misunderstandings of epidemiological data collection, monitoring, and analysis. This approach is also supported by previous work on COVID-19 knowledge-seeking behaviour which is focused more on the technical aspects of the COVID-19 such as symptoms, treatments, and transmission.^24,77^ Press officers can be reassured that, while many of the reports included in our study were heavily caveated due to inconclusive or insufficient evidence available at the time, we did not see a clear pattern that this caveating affected news coverage. This was demonstrated by our finding that the heavily caveated ‘spread by children’ report received a lot of media coverage. This also agrees with previous work that caveats of research did not decrease the likelihood of news coverage or newsworthiness ^83,84^

 From a journalistic standpoint, there were several areas which could be improved upon and selective reporting and inclusion of different stakeholders sometimes changed messages from more evidence-focused to emotion or opinion-focused. Researchers have suggested a list of the minimum requirements needed in PRs announcing COVID-19 clinical trial results.^85^ Building upon previous work which has created tools for improving the quality of health-research based news,^86^ perhaps similar guidance should be created for PRs and journalists. Given the recent increase in the popularity and need for rapid reviews, journalists may be less familiar with reporting these results as opposed to results from primary research studies. Furthermore, as journalists can be equally vulnerable in the event of a viral pandemic, as opposed to reporting on chronic non-infectious diseases, biases and the potential for selective framing.^73^

 Our study has several limitations. Firstly, the print and broadcast media only included news sources which were from the Republic of Ireland and met the criteria for Rue Point Media’s search strategy. News items from Northern Ireland, the rest of the United Kingdom, and other international news outlets were not included. However, as reports were produced to help inform *national *public health responses, we believe this restriction was appropriate, although it is possible that we did not identify all the news stories associated with those PRs. This case study also focuses on a limited corpus of reports during a distinct period of time (April – July 2020). Results cannot necessarily be extended to all reports, nor ones published outside of this time period, especially as recent work has found that media coverage has actually decreased over time, despite the deepening COVID-19 crisis; media coverage rapidly increased in February and March 2020 then steadily decreased.^87^ Another potential bias could have been introduced by the use of one coder (MKS). However, the coding schema and results was continually discussed with another researcher (BC) and all coding was double-coded by a researcher (MKS) who was not involved with the creation of the reports, thus inherent biases may be less. Lastly, our study also focused on traditional media coverage and did not include social media discussions about these reports. Although the prominence of print and broadcast media has declined in recent years, it still represents a significant portion (64% and 32% respectively) of sources of news in Ireland.^88^ Recent research in the US and Canada has also shown that mainstream media (eg, television, radio, podcasts, or newspapers) represents the largest source of information about COVID-19.^21,70^ While it is inarguable that social media plays an important role in the dissemination of information related to COVID-19,^8,11,89,90^ we believe that realm deserves a separate methodology and investigation and is outside the scope of this analysis.

## Conclusion

 From our pool of nearly 500 media sources reporting on nine research reports and four PRs, we found that media coverage largely did not distort or misrepresent the results of the reports, however, there was variability in terms of what content was reported on and to what extent different stakeholders were involved in contextualizing findings. Coverage appeared to focus more on ‘human-interest’ stories as opposed to more technical reports (eg, focusing on viral load, antibodies, testing, etc). Particularly in the ‘human-interest’ reports, there was tension between stakeholders wherein public health and medical experts expressed more cautious interpretations of the evidence versus more enthusiastic responses from the public and politicians. Selective reporting and the variability in the use of quotes from governmental and public health stakeholders changed and contextualized results in different manners than perhaps originally intended in the PR. This emphasises the need for rigour and accuracy in health research related PRs and the need for better engagement with journalists, who are indirect sources of public education, particularly in a pandemic situation.

## Ethical issues

 All data used was publicly available thus ethical approval was not sought.

## Competing interests

 Several co-authors are employees of the Health Information and Quality Authority. Authors declare that they have no other competing interests.

## Authors’ contributions

 Conceptualization: MKS, ZF, CM, MO, MR, and BC; Methodology: MKS, ZF, CM, and BC; Formal analysis and validation: MKS and BC; Resources: ZF, CM, MO, MR, and BC; Data curation: MKS, EO, and BC; Writing–Original Draft: MKS and BC; Writing–Review and Editing: MKS, ZF, CM, EO, SS, MO, MR, and BC; Visualisation: MKS and BC; Supervision: MKS and BC; Project administration: MKS and BC; Funding acquisition: SS, MO, MR, and BC.

## Funding

 Study team members were funded by Health Research Board (HRB) Emerging Investigator Award (EIA-2019-09) and HRB-CICER-2016-1871.

## Supplementary files 

Supplementary file 1. Search Terms Used.
