# Supplementary material for: Irish Media Coverage of COVID-19 Evidence-Based Research Reports From One National Agency
Source: Int J Health Policy Manag. 2021 Dec 13;11(11):2464–75. doi: 10.34172/ijhpm.2021.169 (PMC9818095; doi:10.34172/ijhpm.2021.169)
Supplement: Supplementary file 1 — Search Terms Used. [file ijhpm-11-2464-s001.pdf]

Article title: Irish Media Coverage of COVID-19 Evidence-Based Research Reports From One National Agency

Journal name: International Journal of Health Policy and Management

Authors' information: Melissa K. Sharp[1]*, Zoë Forde[2], Cordelia McGeown[2], Eamon O'Murchu[2], Susan M. Smith[1], Michelle O'Neill[2], Máirín Ryan[2,3], Barbara Clyne[1,2]

[1]Health Research Board Centre for Primary Care Research, Department of General Practice, Royal College of Surgeons in Ireland, Dublin 2, Ireland.

[2]Health Information and Quality Authority, George's Court, George's Lane, Dublin 7, Ireland.

[3]Department of Pharmacology & Therapeutics, Trinity College Dublin, Trinity Health Sciences, Dublin 8, Ireland.

(*Corresponding author: melissasharp@rcsi.com)

Supplementary file 1. Search Terms Used.

National Patient Experience Survey
Health Quality and Information Authority
National Care Experience Programme
HIQA
Health and Information Quality Authority
Phelim Quinn
Máirín Ryan
Mairin Ryan
Mary Dunnion
patient experience survey
National Inpatient Experience Survey
Rachel Flynn
National Maternity Experience Survey
Health Information and Quality Authority
Health Authority for Quality and information
H1QA
HIQUA

===OR===

Health Information and Quality Authority
HIQA

-AND-

concerns
older people
HSE
Minister for Health
ionizing radiation
inspection
digital health
response times
Tusla

HPV
antibiotic
disability
residential service for adults with disabilities
regulation
regulatory powers
National Standards for the Protection and Welfare of Children
ionising radiation
hygiene
antimicrobial
National Standards for Residential Care Settings for Older People in Ireland
nursing home inspection
information governance
Patient Safety Bill
National Ambulance Service
health technology assessment
disabilities
National Standards for Safer Better Healthcare
HTA
eHealth
electronic health record
X-ray
residential service for children with disabilities
child protection
Health Service Executive
notifications
patient safety
Child and Family Agency
Patient Safety Licensing Bill

===OR===

health watchdog
health watchdogs

-AND-

Health Information and Quality Authority
HIQA

Mary Butler
Roderic O'Gorman
Roderic OGorman
Stephen Donnelly
Roderic O Gorman
Anne Rabbitte

-AND-

HIQA
Health Information and Quality Authority
Health Information Quality Authority
